# Development of the quality of teen trauma acute care patient and parent-reported experience measure

**DOI:** 10.1186/s13104-022-06194-x

**Published:** 2022-09-23

**Authors:** Matthew Yeung, Brent E. Hagel, Niklas Bobrovitz, Thomas H. Stelfox, Natalie L. Yanchar

**Affiliations:** 1 Department of Surgery, University of Calgary, Calgary, Canada; 2 Department of Pediatrics, University of Calgary, Calgary, Canada; 3 Department of Critical Care, University of Calgary, Calgary, Canada; 4 Temerty Faculty of Medicine, University of Toronto, Toronto, Canada

**Keywords:** Adolescents, Patient-reported experience measure, Trauma, Trauma care, Injuries, Injury care

## Abstract

**Objective:**

Patient-Reported Experience Measures (PREMs) provide valuable patient feedback on quality of care and have been associated with clinical outcomes. We aimed to test the reliability of a modified adult trauma care PREM instrument delivered to adolescents admitted to hospital for traumatic injuries, and their parents. Modifications included addition of questions reflecting teen-focused constructs on education supports, social network maintenance and family accommodation.

**Results:**

Forty adolescent patients and 40 parents participated. Test-retest reliability was assessed using Cohen’s kappa, weighted kappa, and percent agreement between responses. Directionality of changed responses was noted. Most of the study ran during the COVID-19 pandemic. We established good reliability of questions related to in-hospital and post-discharge communication, clinical and ancillary care and family accommodation. We identified poorer reliability among constructs reflecting experiences that varied from the norm during the pandemic, which included “maintenance of social networks”, “education supports”, “scheduling clinical follow-ups” and “post-discharge supports”. Parents, but not patients, demonstrated more directionality of change of responses by responding with more negative in-hospital and more positive post-discharge experiences over time between the test and retest periods, suggesting risk of recall bias. Situational factors due to the COVID-19 pandemic and potential risks of recall bias may have limited the reliability of some parts of the survey.

**Supplementary Information:**

The online version contains supplementary material available at 10.1186/s13104-022-06194-x.

## Introduction

Patient-reported experience measures (PREMs) and outcome measures (PROMs) can guide alignment of care practices with patient and family preferences, needs, and values [1]. Positive patient and family experiences in medical and surgical care have been shown to improve clinical outcomes, including attention to medication regiments, adverse event reporting, rehabilitative therapy outcomes, and even length of stay (LOS) [2–4]. Patient and family experiences can also influence clinical decision-making, care coordination, and communication, enabling improved clinical outcomes [5]. Previous research highlights the importance of patient experience research in adolescents, who may fall between the patient management approaches of pediatric versus adult centers. For example, adolescents may find themselves in a position where they recognize the need to understand their own healthcare information but are not given the opportunity to do so [6]. As such, several PREMs and PROMs instruments have been developed for children and/or their families that evaluate general experiences of healthcare or focus on specific fields such a juvenile arthritis, pediatric allergies, inflammatory bowel disease, epilepsy, diabetes, cancer, emergency care and mental health [7–9]. PREMs targeting adolescents specifically are uncommon but have been reported for substance use treatment [10].

Despite extensive research in clinical management and outcomes of traumatic injuries in children and adolescents, there has been little research in this growing field of patient and family experience with regards to management of traumatic injuries. We sought to develop an instrument to measure PREMs/PROMs of adolescents and their parents for a proposed comparative analysis of adolescents managed at adult versus pediatric trauma centers (unpublished data). Development and Reliability testing of a new Quality of Teen Trauma Acute Care Patient-Reported Experience Measure (QTTAC-PREM), patient and parent versions, is reported here.

## Main text

### Methods

#### QTTAC-PREM development

We chose to adapt the Short-form version of the Quality of Trauma Acute Care Patient-Reported Experience Measure (QTAC-PREM), developed by Bobrovitz, which focuses on in-hospital and post-discharge phases of adult (16y +) trauma care, and was derived within the same regional trauma system as the proposed comparative study [11, 12]. The QTAC-PREM was developed through an iterative process involving focus groups, cognitive interviews, and pilot testing and found to have moderate to excellent test-retest reliability [11–13]. Several questions were added to the short-form QTAC-PREM to target constructs known to influence adolescent health and wellness. Consultation with adolescent medicine experts and employment of the HEADSS (home, education and employment, activities, drugs, sexuality, suicide/depression) framework identified areas of adolescent health not addressed in the original measure including questions focusing on “Educational supports”, “Social supports” and “Family accommodation” [14]. Questions focusing on post-discharge pain control and receipt of prescriptions for opiates, were included to explore increasing risk of opioid dependency in adolescence [15]. To target parent-specific needs, we queried family accommodation opportunities and use during their child’s hospital admission, adapting from work done previously by Franck [16]. Questions targeting school supports were adapted from the Pediatric Integrated Care Survey, a previously validated PREM examining family experiences of care integration within pediatrics [17]. Questions on maintenance of social support were adapted from the Social Support Questionnaire for Children [18]. Questions were formulated to address the adolescent patient in the patient version and the caregiver in the parent version.

Modifications resulted in 24 and 25 new in-hospital and post-discharge care questions for the patient and parent surveys, respectively. The new survey tool was piloted for readability with a group of adolescents recruited from the Family and Community Resource Centre of the Alberta Children’s Hospital and teen family members of the study team. This informed further modifications to question wording and survey logic, framing questions to be understood by adolescents.

Copies of the Teen and Parent-versions of the QTTAC-PREM are available as supplementary files at Additional files 1 and 2.

#### Reliability testing

Participants for reliability testing of the QTTAC-PREM were recruited as part of a larger study comparing adolescent patient and parent experience at the regional pediatric or adult trauma center (unpublished data). Adolescents (15–17 years) admitted with an injury diagnosis between 01/01/2020 and 31/05/2021 were invited to participate, along with one of their parents. We excluded patients injured from self-inflicted events to avoid confounding from psychological factors that could bias experiences relative to those sustaining non-self-inflicted injuries. We excluded patient whose primary nurse felt they and/or their parent would be unable to provide informed consent or complete the survey due to cognitive impairments (e.g. brain injury or pre-existing deficits) or language barriers (the survey was only available in English). If more than one guardian was with the patient during their hospital stay, the guardian who self-identified as being with the patient the most was chosen for the study.

Based on the proposed sample size of 75 from previous reliability testing of the original QTAC-PREM, we aimed for 80 participants, enrolling the first 40 patients and 40 parents recruited into the larger study agreeing to participate in this retest process [13].

Using emails provided, patients and parents were sent an electronic link to the patient or parent version of the QTTAC-PREM 8 weeks post-discharge, accommodating the assumption that most patients would have had their follow-up care by then. Surveys were completed online using the Qualtrics platform of the University of Calgary. Retesting was done by emailing the same survey 7–10 days after initial survey completion. Participants were offered a $25 gift card for each test and retest survey completed.

#### Data analysis

Cohen’s kappa [with 95% confidence intervals (CI)] and percentage agreement were calculated to determine the level of agreement between test and retest survey responses for each participant. A weighted kappa was used for questions assessed on an ordinal scale. All data analysis was completed in SPSS version 25.

We used Cohen’s suggested values for interpretation, with kappa scores 0.01–0.20 reflecting slight agreement, 0.21–0.40 fair, 0.41–0.60 moderate, 0.61–0.80 substantial, and 0.81–1.00 near-perfect [19]. Though kappa remains a validated and well-used measure of reliability, we observed limitations for questions where responses tended to be uni-modal, with low kappa scores despite very high percentage agreement. Because agreement remained significantly greater than chance and CIs for these questions tended to be large, percentage agreement was chosen to allow for inclusion of such questions, provided their CIs also suggested at least moderate reliability. As such, questions were deemed reliable if they reached a kappa of ≥ 0.41 (moderate or greater agreement) or a percentage agreement of > 80% if kappa CIs exceeded 0.40 as suggested by Miles [19, 20]. Where applicable, descriptive comparisons of kappa values were made with those of the original QTAC-PREM [12].

Directionality of change of responses between the test and the retest phases (reported experience being more negative or positive) was recorded to glean if responses may have changed over time due to changes in overall perceptions of experience, which would be suggested if responses generally changed in one direction versus randomly in either direction. Questions where a greater proportion of retest responses becoming more negative than positive were categorized as “became more negative”; those where a greater proportion became mor positive were categorized as “became more positive”; those with equal proportions were categorized as “no change”.

## Results

Forty adolescents and 40 parents completed the QTTAC-PREM test-retest process, representing 77% and 71%, respectively, of the larger study recruitment. The test-retest reliability scores for individual questions are highlighted in Table 1.

**Table 1:** Survey Test-Retest Kappa and Agreement Percentage

Adolescent patient questions	Re-test agreement (%)	Kappa (poor reliability highlighted)	95% CI	Kappa from original QTAC-PREM	Parent questions	Re-test agreement (%)	Kappa (poor reliability highlighted)	95% CI	Kappa from original QTAC-PREM
PREM questions									
In-hospital information and communication									
Q6.2 How often did your healthcare practitioners clearly explain all your injuries to you in a way you could understand?	64	0.27^A^	-0.03–0.56	0.51	Q7.2 How often did your teen's healthcare practitioners clearly explain all their injuries to you in a way you could understand?	78	*0.44*^*A,B*^	0.16–0.73	0.51
Q6.3 How often did the healthcare practitioners (e.g. doctors, nurses, therapists, etc.) explain what was happening in a way you could understand?^C^	82	*0.61*^*A*^	0.37–0.86		Q7.3 How often did the healthcare practitioners (e.g. doctors, nurses, therapists, etc.) explain your teen's treatment in a way you could understand?^C^	85	*0.63*^*A*^	0.40–0.85	n/a
Q13.1 Did the healthcare practitioners give instructions on how you should care for your injuries?	*82*	0.36^A^	*-0.01–0.73*	0.64	Q14.2 Did the healthcare practitioners give instructions on how you should care for your teen's injuries?	90	*0.57*^*A*^	0.25–0.89	0.64
Q6.4 Did the healthcare practitioners discuss how long it might take you to recover from your injuries?	*85*	0.40	*-0.17–0.97*	0.69	Q7.4 Did the healthcare practitioners discuss how long it might take your teen to recover from their injuries?	93	*0.51*	0.10–0.92	0.69
Q6.5 Did the healthcare practitioners discuss the long-term consequences of your injuries (on sports, music, extracurriculars, etc.) after you leave the hospital?^C^	85	*0.64*	0.40–0.89		Q7.5 Did the healthcare practitioners discuss the long-term consequences of your teen's injuries (on sports, music, extracurriculars, etc.) after they leave the hospital?^C^	90	*0.62*	0.29–0.95	n/a
Q6.6 How often was the information that was given by your various healthcare practitioners consistent?	77	*0.56*^*A*^	0.31–0.80	0.78	Q7.6 How often was the information that was given by your teen's various healthcare practitioners consistent?	83	*0.64*^*A*^	0.38–0.90	0.78
Clinical and ancillary care									
Q6.1 When meeting new healthcare practitioners for the first time, how often did they introduce themselves and clearly explain their role in your care?	79	*0.47*^*A*^	0.17–0.77	0.78	Q7.1 When meeting new healthcare practitioners for the first time, how often did they introduce themselves and clearly explain their role in your teen's care?	*83*	0.37^A^	*0.00–0.74*	0.78
Q84 When the healthcare practitioners helped you to move around (i.e., change position in bed, walking, etc.) how often did they do it carefully?	62	*0.46*^*A*^	0.20–0.71	0.64	Q93 When the healthcare practitioners helped your teen to move around (i.e., change position in bed, walking, etc.) how often did they do it carefully?	61	0.29^A^	0.05–0.54	0.64
Q10.4 How often was the pain from your injuries well controlled?	56	*0.41*^*A*^	0.19–0.62	0.72	Q11.4 How often was your teen's pain from their injuries well controlled?	80	*0.64*^*A*^	0.44–0.85	0.72
Q10.5 How often did the healthcare practitioners do everything they could to help you with your discomfort, agitation or irritability?	77	*0.65*^*A*^	0.46–0.85	0.71	Q11.5 How often did the healthcare practitioners do everything they could to help your teen with their discomfort, agitation or irritability?	80	*0.53*^*A*^	0.26–0.79	0.71
Q10.6 When you had questions, concerns or frustrations about your care, how often did your healthcare practitioners take action?	51	0.30^A^	0.09–0.51	0.68	Q11.6 When you or your teen had questions, concerns or frustrations about your teen's care, how often did their healthcare practitioners take action?	66	0.25^A^	-0.03–0.52	0.68
Q10.7 Did healthcare practitioners (e.g. psychologist, social worker, nurse) offer to speak to you about your mental or emotional health?	59	*0.52*^*A*^	0.30–0.74		Q11.7 Did a healthcare practitioner (e.g. psychologist, social worker, nurse) offer to speak to you or your teen about their mental or emotional health?	61	*0.59*^*A*^	0.40–0.79	n/a
Q10.8 How often did the hospital staff offer to help you maintain your personal hygiene (brushing teeth, bathing, etc.)?	62	*0.58*^*A*^	0.37–0.79	0.68	Q11.8 How often did the hospital staff offer to help your teen maintain their personal hygiene (brushing teeth, bathing, etc.)?	61	*0.68*^*A*^	0.52–0.83	0.68
Q10.10 How often did you experience care that you thought was unsafe?	*87*	0.22^A^	*-0.15–0.59*	0.88	Q11.10 How often did your teen experience care that you thought was unsafe?	100	*1.00*		0.88
Q10.11 How often were you treated unfairly because of your age, ethnicity, gender, or personal characteristics?	100	*1.00*		0.44	Q11.11 How often was your teen treated unfairly because of their age, ethnicity, gender, or personal characteristics?	100	*1.00*		0.44
Q10.12 How often did you feel you were treated in a way that was not appropriate for your age?^C^	*90*	0.23^A^	*-0.17–0.63*		Q11.12 How often did you feel your teen was treated in a way that was not appropriate for their age?^C^	100	*1.00*		n/a
Social network supports									
Q8.1 How often did your friends visit you while you were in hospital?^C^	82	*0.65*^*A*^	0.41–0.89		Q9.1 How often did your teen's friends visit them while they were in hospital?^C^	85	*0.81*^*A*^	0.66–0.97	n/a
Q8.2 Why do you think your friends did not visit you in hospital (select all that apply)?^C^	95	*0.74*	0.42–1.07		Q9.2 Why do you think your teen's friends did not visit them in hospital (select all that apply)?^C^	53	0.40	0.16–0.64	n/a
Q8.3 How often do you think your friends would have felt comfortable visiting you while you were in the hospital?^C^	52	0.32^A^	0.04–0.59		Q9.3 How often do you think your teen's friends would have felt comfortable visiting them while they were in hospital?^C^	47	0.21^A^	-0.05–0.46	n/a
Q8.4 How often would you have felt comfortable having your friends visit you while you were in the hospital?^C^	52	0.18^A^	-0.08–0.45		Q9.4 How often would your teen have felt comfortable having their friends visit them while they were in the hospital?^C^	86	*0.77*^*A*^	0.47–1,08	n/a
Q8.5 How often do you think your friends felt comfortable visiting you while you were in the hospital?^C^	75	*0.62*^*A*^	0.09–1.14		Q9.5 How often do you think your teen's friends felt comfortable visiting them while they were in hospital?^C^	71	*0.56*^*A*^	0.16–0.96	n/a
Q8.6 How often did you feel comfortable having your friends visit you while you were in the hospital?^C^	75	*0.43*^*A*^	0.080.78		Q9.6 How often do you think your teen felt comfortable having their friends visit them while they were in the hospital?^C^	76	*0.56*^*A*^	0.35–0.77	n/a
Education supports									
Q11.2 How often did your healthcare practitioners help you to keep up with schoolwork while in hospital?^C^	*83*	0.18^A^	*-0.21–0.58*		Q12.2 How often did your teen's healthcare practitioners help them to keep up with schoolwork while in hospital?^C^	86	*0.50*^*A*^	0.10–0.89	n/a
Q11.3 How often did your healthcare practitioners ask if you required extra services for schoolwork related to your injures (i.e. someone to write for you, extra time on tests)?^C^	78	0.33^A^	-0.02–0.69		Q12.3 How often did your teen's healthcare practitioners ask if they required supplemental services for schoolwork related to their injuries(i.e. someone to write for them, extra time on tests)?^C^	81	*0.51*^*A*^	0.16–0.86	n/a
Family accommodation									
Q7.1 During your stay in hospital, were accommodations available for one or more of your parents to stay with or near you (e.g. down the hall, a bed in your room)?^C^	92	*0.47*	0.10–0.84		Q8.2 Were accommodations available for you/the teen's caregiver to stay with or near your teen (e.g. down the hall, a bed in your teen's hospital room)?^C^	88	*0.49*	0.30–0.69	n/a
Q7.2 During your stay in hospital, how often did one or more of your parents/caregivers stay overnight with or near you?^C^	82	*0.80*^*A*^	0.64–0.96		Q8.3 During your stay in hospital, did you or another caregiver stay overnight with or near your teen?^C^	98	*0.93*^*A*^	0.83–1.03	n/a
Q10.9 Did you have privacy in your hospital room?^C^	69	*0.55*^*A*^	0.33–0.78		Q11.9 Did you have privacy in your teen's hospital room?^C^	59	*0.58*^*A*^	0.40–0.76	
Global rating of in-hospital care									
Q12.1 Please provide an overall rating, between 0 and 10, of the hospital care you received for your injury (with 0 being the worst care possible and 10 being the best)?	62	*0.55*^*A*^	0.36–0.74	0.85	Q13.1 Please provide an overall rating, between 0 and 10, of the hospital care they received for their injury (with 0 being the worst care possible and 10 being the best)?	71	*0.60*^*A*^	0.37–0.82	0.85
Scheduling follow-ups									
Q17.1 After being discharged from the hospital, have you attended an appointment to follow-up about your injuries with… (select all that apply)^C^	77	*0.69*	0.52–0.86		Q18.1 After being discharged from the hospital, did your teen attend any appointments to follow-up about their injuries with… (select all that apply)^C^	73	*0.65*	0.48–0.82	n/a
Q17.2 Have you scheduled or are planning to schedule an appointment to follow-up about your injuries with… (select all that apply)^C^	54	*0.45*	0.27–0.63		Q87 Have you or your teen scheduled or are planning to schedule an appointment to follow-up about their injuries with… (select all that apply) ^C^	63	0.40	0.26–0.55	n/a
Q78 Have you had difficulty scheduling an appointment to follow-up about your injuries with… (select all that apply)	*87*	0.40	*-0.02–0.82*		Q18.2 Did you have any difficulty scheduling follow-up appointments for your teen when either of you wanted them with… (select all that apply)	80	0.27	-0.01–0.55	n/a
Discharge and post-discharge information and communication									
Q13.2 Before leaving the hospital, did your doctors or nurses give you or your parents written instructions on how to care for your injuries after being discharged?^D^	79	*0.52*	0.27–0.77	0.78	Q14.3 Before leaving the hospital, did your teen's doctors or nurses give you or another caregiver written instructions on how to care for their injuries after being discharged?^D^	87	*0.49*	0.10–0.88	0.78
Q13.3 Did the written instructions provided give you enough information to help you take care of your injuries after being discharged?^D^	100	*1.00*			Q14.4 Did the written instructions provided give him/her enough information to help him/her to care for his/her injuries after being discharged?^D^	100	*1.00*		n/a
Q17.5 At your follow-up appointments, did your healthcare practitioners explain the next steps in your recovery from your injury? For example, activities you should or should not do, necessary medications, tests, treatments, or other follow-up appointments?	71	0.23	-0.07–0.53	0.71	Q18.5 At your teen's follow-up appointments, did your healthcare practitioners explain the next steps in your teen's recovery from his/her injury; for example, activities they should or should not do, necessary medications, tests, treatments, or other follow-up appointments?	*85*	0.39	*0.05–0.73*	0.71
Q79 At your follow-up appointments, did your healthcare practitioners explain approximately how long it would take you to recover?	76	0.25	-0.04–0.53	0.55	Q88 At your teen's follow-up appointments, did their healthcare practitioners explain approximately how long it would take them to recover?	90	*0.71*	0.47–0.94	0.55
Q17.6 At your follow-up appointments, how often did your healthcare practitioners explain things about your injuries in a way that you could understand?	76	*0.48*^*A*^	0.25–0.70	0.69	Q18.6 At your teen's follow-up appointments, how often did your healthcare practitioners explain things about his/her injuries in a way that you and your teen could understand?	88	*0.66*^*A*^	0.41–0.91	0.69
Q19.1 Overall, how well were you guided through your recovery process by your healthcare providers after being discharged from the hospital (on a scale of zero to ten, zero being poor guidance, ten being excellent guidance)?^C^	54	*0.56*^*A*^	0.37–0.74		Q20.1 Overall, how well were you and your teen guided through the recovery process by his/her healthcare practitioners after being discharged from the hospital on a scale of zero to ten (zero being poor guidance, ten being excellent guidance)?^C^	68	*0.71*^*A*^	0.57–0.85	n/a
Discharge and post-discharge supports									
Q16.1 After being discharged from the hospital, did you get all of the support services that you wanted or felt you needed? (for example, home care, social work, or counselling)^C^	*85*	0.38	*0.03–0.73*		Q17.1 After being discharged from the hospital, did your teen get all of the support services that they wanted or felt they needed? (for example, home care, social work or counselling)^C^	78	*0.44*	0.22–0.65	n/a
Q17.7 At your follow-up appointments, when you or your parents expressed concerns or frustrations, how often did your healthcare practitioners take action to deal with them?^C^	55	0.24^A^	-0.01–0.48		Q18.7 At your teen's follow-up appointments, when you or your teen expressed concerns or frustrations, how often did his/her healthcare practitioners take action to deal with them?^C^	61	0.38^A^	0.13–0.62	n/a
Q17.8 At your follow-up appointments, were school attendance and/or performance issues specifically addressed by any of the healthcare practitioners?^C^	63	0.15^A^	-0.18–0.49		Q18.8 At your teen's follow-up appointments, were school attendance and/or performance issues specifically addressed by any of the healthcare practitioners?^C^	71	*0.54*^*A*^	0.30–0.78	n/a
Q17.9 At your follow-up appointments, how often did your healthcare practitioners ask if you required extra services for schoolwork (i.e. someone to write for you, extra time on tests), related to your injuries?^C^	82	*0.61*^*A*^	0.34–0.89		Q18.9 At your teen's follow-up appointments, how often did your teen's healthcare practitioners ask if they required extra services for schoolwork (i.e. someone to write for them, extra time on tests), related to their injuries?^C^	88	*0.76*^*A*^	0.53–0.98	n/a
Post-discharge pain control									
Q15.1After being discharged from the hospital, did you have enough pain medication to control your pain well?	82	*0.65*	0.42–0.89	0.90	Q16.1 After being discharged from the hospital, did your teen have enough pain medication to control his/her pain well?	83	*0.63*	0.38–0.88	0.90
Communication with primary care									
Q80 Did your family physician, pediatrician or general practitioner receive information from the hospital about your injures, your hospital stay, or the care you would need to continue your recovery?	72	*0.61*	0.41–0.80	0.87	Q89 Did your teen's family physician, pediatrician, or general practitioner receive information from the hospital about your injures, your hospital stay, or the care you would need to continue your recovery?	73	*0.62*	0.45–0.80	0.87
Global rating of post-discharge care									
Q19.2 On a scale of zero to ten, what is your overall rating of the follow-up care you received after being discharged from the hospital (zero being the worst injury care possible, ten being the best)?	54	*0.56 *^*A*^	0.39–0.74		Q20.2 On a scale of zero to ten, please provide an overall rating of the follow-up care your teen received after being discharged from the hospital (zero being the worst injury care possible, ten being the best)?	80	*0.76*^*A*^	0.62–0.91	n/a
Additional non-PREM questions									
Q15.2 After being discharged from the hospital, did you receive a prescription for opioids to control your pain? (Ex. Tramadol, Dilaudid, Ultram, etc.)^C^	79	*0.72*	0.49–0.95		Q16.2 After being discharged from the hospital, did your teen receive a prescription for opioids to control his/her pain? (Ex. Tramadol, Dilaudid, Ultram, etc.)^C^	81	*0.78*	0.56–1.00	n/a
Q15.3 After being discharged from the hospital, did you fill out your prescription for opioids to control your pain? (Ex. Tramadol, Dilaudid, Ultram, etc.)^C^	100	*1.00*			Q16.3 After being discharged from the hospital, did your teen fill out your prescription for opioids to control his/her pain? (Ex. Tramadol, Dilaudid, Ultram, etc.)^C^	78	*0.71*	0.21–1.22	n/a
Q9.1 How did you communicate with your friends during your stay in hospital, the majority of the time?^C^	90	*0.81*	0.63–0.98						
					Q8.1 Which best describes the overnight accommodation you used most during your teen's hospital stay?^C^	83	*0.68*	0.48–0.88	n/a

Questions with shaded statistics test as unreliable

^A^Weighted Kappa Calculated

^B^Statistics in italics represent significant measure of reliability

*n/a* not applicable

^C^Added question compared to QTAC-PREM SF

^D^Questions were originally a singular question in original QTAC-PREM

Kappa scores of adolescents ranged between 0.18 and 1.0. with 30/46 questions from the patient version reaching moderate- to near-perfect reliability. A further seven questions were considered reliable with response agreements being > 80% and kappa 95% CIs spanning > 0.40.

Amongst adolescents, nine questions with poor reliability predominated in the PREM constructs of “Social network supports”, “Education supports”, “Discharge and post-discharge information and communication” and “Post-discharge supports”. Isolated questions from each of “In-hospital information and communication” and “Clinical and ancillary care” constructs also tested as unreliable, although appeared reliable with patients > 15 years old in the original QTAC-PREM [12]. For both in-patient and post-discharge care, questions about healthcare responses to expression of frustrations were unreliable. All other constructs, global ratings and isolated questions tested as reliable.

Amongst parents, seven questions with poor reliability predominated in the PREM constructs of “Social network supports” and “Scheduling follow-ups”. Isolated questions from each of “Clinical and ancillary care” and “Post-discharge supports” also tested as unreliable, although appeared reliable with patients > 15 years old in the original QTAC-PREM [12]. As with adolescents, questions asking about healthcare responses to expression of frustrations whether in-hospital or post-discharge were unreliable. All other constructs and global rating questions tested as reliable.

**Fig. 1:**
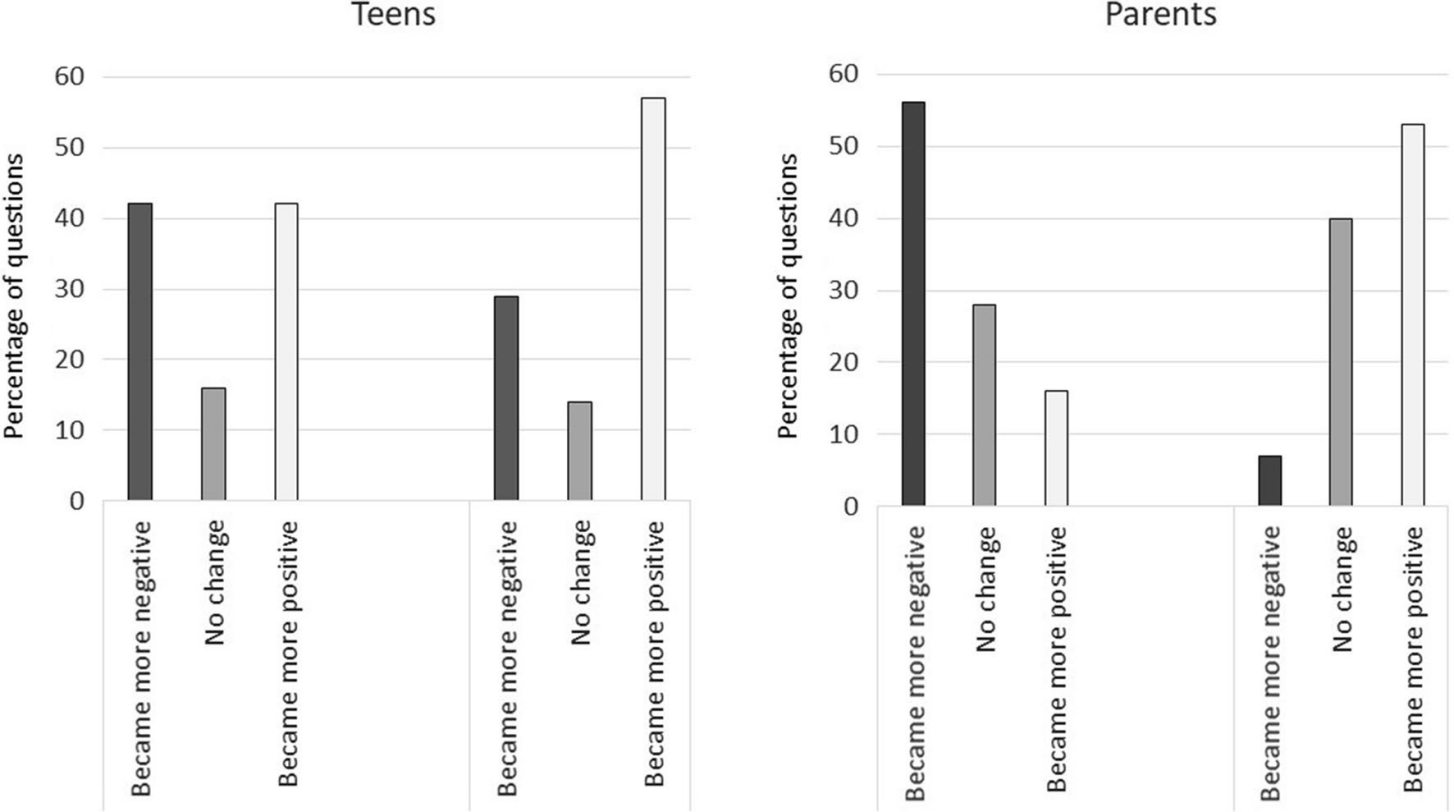
Direction of change of responses between test and retest phases

There was little consistency of directionality of changes in responses between the test and retest phases among adolescents (Fig. 1). Amongst parents, however, there tended to be an increase of reporting negative in-hospital experiences and positive post-discharge experiences over the test-retest timeframe.

## Discussion

Variable reliability of components of the QTTAC-PREM was observed when applied to hospitalized injured adolescent patients and their parents. These observations can be attributed to situational and survey administration factors.

Many patients only stayed 1 or 2 days in hospital making the need for education supports inconsequential. In addition, 13 of the 17 months over which this study was run was during the SARS-CoV-2 (COVID-19) pandemic, when on-line schooling prevailed for many schools in the trauma centers’ catchment areas. These two situations likely resulted in questions related to school support needs less relevant to all respondents; superficial reflection on these could thereby make them prone to poor test-retest results. Alternatively, needs for outside supports for post-discharge issues such a homecare, and dealing with frustration and school performance issues, may have been compounded by limited access to supports because of the pandemic, confounding responses between the test and retest phases. Similarly, poor reliability on questions related to social network supports may have affected questions related to having friends visit. During the pandemic, visitors were extremely limited in acute care settings, likely rendering these questions inconsequential and subject to inference rather than true experience.

Asking questions pertaining to in-hospital care, eight weeks post-discharge, may also have decreased their reliability due to respondent recall, Reliability of acute care questions in the original (unmodified) QTAC-PREM was relatively strong, with reliability coefficients ranging between 0.44 and 0.88 [12]. Unlike our study, these were tested at the end of patients’ hospital stays, albeit with only a 24-h lag between the test and re-test processes [11]. This original PREM instrument primarily focused on only the two constructs of clinical and ancillary care and information and communication. Although not as strong as the original PREM instrument, these two constructs still remailed mostly reliable in our study, despite the 8-week lag post-discharge, suggesting that the 8-week lag post-discharge may only partially explain any recall bias.

Amongst parent responses, a stronger directionality, namely in-hospital experiences being reported as more negative and post-discharge experiences as more positive suggest that overall perceptions of these two phases may have changed somewhat overtime, with a resultant overall influence on reliability in the retesting phase of parents. On the contrary, the lack of clear overall directional change of responses over time with the adolescent group may suggest more randomness of their retest responses. Similarly, poor reliability of questions pertaining to post-discharge supports and information/communication may be related to timing of survey administration if some patient completed the first survey prior to follow-up with their health care practitioner and the re-test after follow-up.

## Recommendations and future directions

Given the limitations outlined above and, notably, the situational factors that may have influenced the test-retest process (COVID-19 pandemic), we would still recommend use of the QTTAC-PREM for examining adolescent and parent experiences during in-hospital and post-discharge care of adolescents hospitalized for traumatic injuries. Retesting of reliability of sections pertaining to maintenance of social networks and schooling reliability and supports needed post-discharge, when there is no concurrent pandemic, may be of value. We also recommend it be delivered in two phases temporal to the in-hospital and post-discharge phases of care, to avoid recall bias.

## Limitations

The COVID-19 pandemic likely influenced the interpretation of some of the questions related to supports for schooling and maintenance of social networks, suggesting need for repeat reliability testing outside the pandemic.

The potential for recall bias must also be acknowledged as the survey inquired about in-hospital care but was administered at eight weeks post-discharge. This 8-week period was chosen with an assumption that most patients would have had their follow-up care within this period. However, the effects of the pandemic on scheduling follow-up appointments may have made this assumption invalid and we would suggest future querying of post-discharge experiences include a screening item to exclude respondents who have not yet had follow-up care. Finally, administering survey questions relating to in-hospital versus post-discharge care at time frames more temporal to these phases of care should be considered.

Our reliability study may also have been limited by our sample size. We note the original study examining the reliability of the QTAC-PREM SF included 117 retest cases, with a calculated sample size requirement of 75 [12, 13]. We were only able to recruit 40 patients and 40 parents and although there were similarities in reliability results between the two respondent groups, variations in directionality suggest that they may not be comparable.

## Supplementary Information

**Additional file 1.** QTTAC-PREOM SF Teen.

**Additional file 2.** QTTAC-PREOM Parent SF.

## Data Availability

Copies of the teen and parent versions of the QTTAC-PREM survey instrument are available in the supplementary files at Additional file 2. The dataset generated and analysed during this study are not publicly available to maintain the anonymity of the research participants and in compliance with the confidentiality statements on the signed consent forms and adherence to ethical approval obtained for the study. Additional data may be available from the corresponding author on reasonable request.

## Abbreviations

PREM: Patient-reported experience measure
PROM: Patient-reported outcome measure
QTTAC-PREM: Quality of teen trauma acute care patient-reported experience measure
QTAC-PREM: Quality of trauma acute care patient-reported experience measure
HEADSS: Home, education and employment, activities, drugs, sexuality, suicide/depression
CI: Confidence interval
