# Supplementary material for: Development of the quality of teen trauma acute care patient and parent-reported experience measure
Source: BMC Res Notes. 2022 Sep 23;15:304. doi: 10.1186/s13104-022-06194-x (PMC9503226; doi:10.1186/s13104-022-06194-x)
Supplement: Supplementary file 1 — Additional file 1. QTTAC-PREOM SF Teen. [file 13104_2022_6194_MOESM1_ESM.pdf]

QTTAC-PREOM SF Teen

Start of Block: Survey Intro

Q1.1
We are doing a research study on teens who got injured and were admitted to a hospital in Calgary over the past year. Our hospital records show that you were one of those teens. We are interested in finding out what you thought of the care you received in hospital and after you were discharged from hospital.

We already contacted your parents and they gave us the OK to email you with this invitation to complete a list of questions online, relating to when you were hospitalized for your injury over the past year. We understand that these injuries occurred some time ago, so we know that some answers may be hard to remember, and that is OK. We are interested in knowing what you think now, as you reflect back on the experience.

You don't have to agree to do this survey if you don't want to. However, we hope you do as we want to use the results of this study to find ways to improve the care of teens, like you, who get seriously injured and have to be hospitalized.

There are about 60 multiple-choice questions, and we have made them as simple as possible. It should take approximately 25 minutes or less. If there is a question you are unsure of the answer, there will be an option to reply "I don't know" or "not applicable." Though it is encouraged, you do not have to complete the entire survey at once. Survey completion is indicated at the end of the survey by text reading "thank you for your participation."

All of your answers will be kept strictly confidential and will only be used for the purpose of this research project. No one except yourself and the researchers will know you participated in this survey.

Q1.2
If you are still okay with going ahead, then please choose "Start survey." If you don't want to participate, then choose "I don't want to participate."

Click the arrow at the bottom right of your screen to proceed.

- ○ Start survey (1)
- ○ I don't want to participate (2)

End of Block: Survey Intro

Start of Block: Randomizer init

Q2.1
Thanks for agreeing to participate in this survey.

By agreeing to do so, this indicates to us that you understand the purpose of the survey and agree to let us use your answers to help find ways we can improve the care of teens who get major injuries and have to be hospitalized.

At the end of the survey, we will ask you for permission to send you an e-gift card as a token of thanks for helping us with this study.

Lets get started!

Click the arrow at the bottom right of your screen to proceed.

End of Block: Randomizer init

Start of Block: PedsQL Block

Q5.1
You are now beginning the Quality of Teen Trauma Care Patient Reported Experience Measure (QTTAC-PREM).

The following questions are about your experience while you were hospitalized, and your experience after you left the hospital.

End of Block: QTAC PREOM Start

Start of Block: Caregiver Accommodation

Q7.1 **During your stay in hospital, were accommodations available for one or more of your parents to stay with or near you (e.g. down the hall, a bed in your room)?**

○ Yes (1)

○ No (2)

○ I don't know/don't remember (3)

Use Reusable Choices

Q7.2 **During your stay in hospital, how often did one or more of your parents/caregivers stay overnight with or near you?**

○ Never (1)

○ Sometimes (2)

○ Usually (3)

○ Always (4)

○ I don't remember/don't know (5)

End of Block: Caregiver Accommodation

Start of Block: Friends block

Use Reusable Choices

Q8.1 **How often did your friends visit you while you were in hospital?**

- ○ Never (1)
- ○ Sometimes (2)
- ○ Usually (3)
- ○ Always (4)
- ○ I don't remember/don't know (5)

Display This Question:

If If How often did your friends visit you while you were in hospital? Never Is Selected

Q8.2 **Why do you think your friends did not visit you in hospital (select all that apply)?**

- ☐ I did not stay in hospital very long (1)
- ☐ They live far from the hospital (2)
- ☐ I told them not to visit me (3)
- ☐ They felt uncomfortable visiting me (4)
- ☐ Other (please describe) (5) ________________
- ☐ I don't remember/don't know (6)

Display This Question:

If If How often did your friends visit you while you were in hospital? Never Is Selected

Or Or How often did your friends visit you while you were in hospital? I don't remember/don't know Is Selected

Use Reusable Choices

Q8.3 **How often do you think <u>your friends would have felt comfortable</u> visiting you while you were in the hospital?**

- Never (1)
- Sometimes (2)
- Usually (3)
- Always (4)
- I don't remember/don't know (5)

Display This Question:

If If How often did your friends visit you while you were in hospital? Never Is Selected

Or Or How often did your friends visit you while you were in hospital? I don't remember/don't know Is Selected

Use Reusable Choices

Q8.4 **How <u>often would you have felt com</u>fortable having your friends visit you while you were in the hospital?**

- Never (1)
- Sometimes (2)
- Usually (3)
- Always (4)
- I don't remember/don't know (5)

Display This Question:

If If How often did your friends visit you while you were in hospital? Never Is Not Selected

And And How often did your friends visit you while you were in hospital? I don't remember/don't know Is Not Selected

Use Reusable Choices

Q8.5 **How often do you think <u>your friends felt comfortable</u> visiting you while you were in the hospital?**

- ○ Never (1)
- ○ Sometimes (2)
- ○ Usually (3)
- ○ Always (4)
- ○ I don't remember/don't know (5)

Display This Question:

If If How often did your friends visit you while you were in hospital? Never Is Not Selected

And And How often did your friends visit you while you were in hospital? I don't remember/don't know Is Not Selected

Use Reusable Choices

Q8.6 **How often did you feel <u>comfortable</u> having your friends visit you while you were in the hospital?**

- ○ Never (1)
- ○ Sometimes (2)
- ○ Usually (3)
- ○ Always (4)
- ○ I don't remember/don't know (5)

Display This Question:

If If How often do you think your friends felt comfortable visiting you while you were in the hospital? Never Is Selected

Or Or How often do you think your friends felt comfortable visiting you while you were in the hospital? Sometimes Is Selected

Or Or How often do you think your friends felt comfortable visiting you while you were in the hospital? Usually Is Selected

Or Or How often do you think your friends felt comfortable visiting you while you were in the hospital? Always Is Selected

Q8.7 **Please tell us why, if ever, you felt <u>your friends</u> were <u>not comfortable</u> visiting you while you were in hospital?**

____

Display This Question:

If If How often did you feel comfortable having your friends visit you while you were in the hospital? Never Is Selected

Or Or How often did you feel comfortable having your friends visit you while you were in the hospital? Sometimes Is Selected

Or Or How often did you feel comfortable having your friends visit you while you were in the hospital? Usually Is Selected

Or Or How often did you feel comfortable having your friends visit you while you were in the hospital? Always Is Selected

Q8.8 **Please tell us why, if ever, did <u>you</u> feel <u>not comfortable</u> with friends visiting you while you were in hospital?**

____

End of Block: Friends block

Start of Block: Friends Block Cont'd

Q9.1 **How did you <u>communicate with your friends</u> during your stay in hospital, the majority of the time?**

- In-person (1)
- Telephone conversations (2)
- Texting (3)
- Social media (Instagram, Snapchat, etc.) (4)
- I was unable to communicate with friends while in hospital (5)
- Other (please explain) (6) ____
- I don't remember/don't know (7)

End of Block: Friends Block Cont'd

Start of Block: School Assistance Block

Q11.1 **What grade in school were you when you were injured?**

- 8th grade or younger (1)
- Grade 9 (2)
- Grade 10 (3)
- Grade 11 (4)
- Grade 12 (5)
- 1st year University or above (6)
- Trade school (7)
- Was not enrolled in school at the time (8)
- I don't remember/don't know (9)

Display This Question:

If What grade in school were you when you were injured? != Was not enrolled in school at the time

Q17.3 **During your hospitalization, approximately how many days of school did you miss?**

- 0-5 (1)
- 6-10 (2)
- 11-15 (3)
- 16-35 (4)
- I am not back at school (5)
- I was not enrolled in school (6)
- I don't know/don't remember (7)

Display This Question:

If What grade in school were you when you were injured? != Was not enrolled in school at the time

Use Reusable Choices

Q11.2 **How often did your healthcare practitioners help you to keep up with schoolwork while in hospital?**

- Never (1)
- Sometimes (2)
- Usually (3)
- Always (4)
- I don't remember/don't know (5)
- Schoolwork was not a concern (6)

Display This Question:

If What grade in school were you when you were injured? != Was not enrolled in school at the time

Use Reusable Choices

Q11.3 **How often did your healthcare practitioners ask if you required extra services for schoolwork related to your injures (i.e. someone to write for you, extra time on tests)?**

- Never (1)
- Sometimes (2)
- Usually (3)
- Always (4)
- I don't remember/don't know (5)
- Schoolwork was not a concern (6)

End of Block: School Assistance Block

Start of Block: Default Question Block

Q6.1 **When meeting new healthcare practitioners for the first time, how often did they introduce themselves and clearly explain their role in your care?**

- Never (1)
- Sometimes (2)
- Usually (3)
- Always (4)
- I don't remember/don't know (5)

Q6.2
How often did your healthcare practitioners clearly explain all your injuries to you in a way you could understand?

- Never (1)
- Sometimes (2)
- Usually (3)
- Always (4)
- I don't remember/don't know (5)

Q6.3 **How often did the healthcare practitioners (e.g. doctors, nurses, therapists, etc.) <u>explain your treatment</u> in a way you could <u>understand</u>?**

- Never (1)
- Sometimes (2)
- Usually (3)
- Always (4)
- I don't know/don't remember (5)

Q13.1 **Did the healthcare practitioners give <u>instructions</u> on how you should <u>care for your injuries</u>?**

- Yes, I received all the instruction I needed (1)
- Yes, but I only received some of the instruction I needed (2)
- No (3)
- I don't remember/don't know (4)

Q6.4 **Did the healthcare practitioners discuss <u>how long</u> it might take you <u>to recover</u> from your injuries?**

- Yes (1)
- No (2)
- I don't know/don't remember (3)

Q6.5 **Did the healthcare practitioners discuss <u>the long-term consequences of your injuries</u> (on sports, music, extracurriculars, etc.) after you leave the hospital?**

- Yes (1)
- No (2)
- I don't know/don't remember (3)

Use Reusable Choices

Q6.6 **How often was the information that was given by your various healthcare practitioners consistent?**

- ○ Never (1)
- ○ Sometimes (2)
- ○ Usually (3)
- ○ Always (4)
- ○ I don't remember/don't know (5)

End of Block: Default Question Block

Start of Block: Block 4

X→

Q84 **When the healthcare practitioners helped you to move around (i.e., change position in bed, walking, etc.) how often did they do it carefully?**

- ○ Never (1)
- ○ Sometimes (2)
- ○ Usually (3)
- ○ Always (4)
- ○ I did not need help moving (5)
- ○ I don't remember/don't know (6)

Q10.4 **How often was the pain from your injuries well controlled?**

- ○ Never (1)
- ○ Sometimes (2)
- ○ Usually (3)
- ○ Always (4)
- ○ I had no pain (5)
- ○ I don't remember/don't know (6)

Q10.5 **How often did the healthcare practitioners do everything they could to help you with your discomfort, agitation or irritability?**

- ○ Never (1)
- ○ Sometimes (2)
- ○ Usually (3)
- ○ Always (4)
- ○ I had no agitation or irritability (5)
- ○ I don't remember/don't know (6)

Q10.6 **When you had <u>questions</u>, <u>concerns</u> or <u>frustrations</u> about your care, how often did your healthcare practitioners <u>take action</u>?**

- ◯ Never (1)
- ◯ Sometimes (2)
- ◯ Usually (3)
- ◯ Always (4)
- ◯ I had no questions, concerns or frustrations about my care (5)
- ◯ I don't remember/don't know (6)

Q10.7 **Did healthcare practitioners (e.g. psychologist, social worker, nurse) <u>offer</u> to <u>speak</u> to you about your <u>mental or emotional health</u>?**

- ◯ Yes, and I got all the support I needed (1)
- ◯ Yes, but I needed more support (2)
- ◯ Yes, but I did not need support (3)
- ◯ No, and I felt I needed support (4)
- ◯ No, but I did not need support (5)
- ◯ I don't remember/don't know (6)

Use Reusable Choices

Q10.8 **How often did the hospital staff <u>offer to help you</u> maintain your <u>personal hygiene (brushing teeth, bathing, etc)</u>?**

- ◯ Never (1)
- ◯ Sometimes (2)
- ◯ Usually (3)
- ◯ Always (4)
- ◯ I don't remember/don't know (5)

Use Reusable Choices

Q10.9 **Did you have <u>privacy</u> in your hospital room?**

- ◯ Never (1)
- ◯ Sometimes (2)
- ◯ Usually (3)
- ◯ Always (4)
- ◯ I don't remember/don't know (5)

Use Reusable Choices

Q10.10 **How often did you experience care that you thought was <u>unsafe?</u>**

- ○ Never (1)
- ○ Sometimes (2)
- ○ Usually (3)
- ○ Always (4)
- ○ I don't remember/don't know (5)

Use Reusable Choices

Q10.11 **How often were you treated <u>unfairly</u> because of your <u>age, ethnicity, gender, or personal characteristics</u>?**

- ○ Never (1)
- ○ Sometimes (2)
- ○ Usually (3)
- ○ Always (4)
- ○ I don't remember/don't know (5)

Use Reusable Choices

Q10.12 **How often did you feel you were treated in a way that was <u>not appropriate for your age</u>?**

○ Never (1)

○ Sometimes (2)

○ Usually (3)

○ Always (4)

○ I don't remember/don't know (5)

End of Block: Block 4

Start of Block: Overall care

Q12.1 **Please provide an overall rating, between 0 and 10, of the hospital care you received for your injury (with 0 being the worst care possible and 10 being the best)?**

○ 0 Worst Injury Care Possible (1)

○ 1 (2)

○ 2 (3)

○ 3 (4)

○ 4 (5)

○ 5 (6)

○ 6 (7)

○ 7 (8)

○ 8 (9)

○ 9 (10)

○ 10 Best injury Care Possible (11)

End of Block: Overall care

Start of Block: Pre-Discharge Care

Q13.2 **Before leaving the hospital, did your *doctors or nurses* give you or your parents written instructions on how to care for your injuries after being discharged?**

○ Yes (1)

○ No (2)

○ I don't remember/don't know (3)

Display This Question:

If Before leaving the hospital, did your doctors or nurses give you or your parents written instruct... = Yes

Q13.3 **Did the written instructions provided give you enough information to help you take care of your injuries after being discharged?**

○ Yes (1)

○ No (2)

○ I don't remember/don't know (3)

End of Block: Pre-Discharge Care

Start of Block: Post-discharge block

Q14.1 **After your stay in hospital…**

End of Block: Post-discharge block

Start of Block: Medications block

Q15.1 **After being discharged from the hospital, did *you* have <u>enough pain medication</u> to <u>control your pain well</u>?**

- ◯ Yes (1)
- ◯ No (2)
- ◯ I didn't need any pain medication (3)
- ◯ I don't remember/don't know (4)

Display This Question:

If After being discharged from the hospital, did you have enough pain medication to control your pa... = Yes

Or After being discharged from the hospital, did you have enough pain medication to control your pa... = No

Or After being discharged from the hospital, did you have enough pain medication to control your pa... = I don't remember/don't know

Q15.2 **After being discharged from the hospital, did *you* <u>receive a prescription for opioids to control your pain?</u> (Ex. Tramadol, Dilaudid, Ultram, etc.)**

- ◯ Yes (1)
- ◯ No (2)
- ◯ I don't remember/don't know (3)

Display This Question:

If After being discharged from the hospital, did you receive a prescription for opioids to control... = Yes

Q15.3 **After being discharged from the hospital, did *you* <u>fill out your prescription for opioids to control your pain?</u> (Ex. Tramadol, Dilaudid, Ultram, etc.)**

- ◯ Yes (1)
- ◯ No (2)
- ◯ I don't remember/don't know (3)

End of Block: Medications block

Start of Block: Post-discharge support services

Q16.1 **After being discharged from the hospital, did *you* get <u>all of the support services that you wanted or felt you needed</u>? (for example, home care, social work, or counselling)**

- ○ Yes (1)
- ○ No (2)
- ○ I don't know/don't remember (3)

Q16.2 **After being discharged, to what extent were you able to maintain your friend/social network?**

- ○ Never (1)
- ○ Sometimes (2)
- ○ Usually (3)
- ○ Always (4)
- ○ I don't know/don't remember (5)

End of Block: Post-discharge support services

Start of Block: Follow-up block

Q17.1 **After being discharged from the hospital, have you attended an appointment to follow-up about your injuries with… (select all that apply)**

- ▢ A trauma doctor, surgeon, or specialist (1)
- ▢ A family doctor (2)
- ▢ A physio, rehabilitation, or occupational therapist (3)
- ▢ Other (please describe) (4) ____
- ▢ None (5)
- ▢ I don't know/don't remember (6)

Q17.2 **Have you scheduled or are planning to schedule an appointment to follow-up about your injuries with… (select all that apply)**

- ▢ A trauma doctor, surgeon, or specialist (1)
- ▢ A family doctor (2)
- ▢ A physio, rehabilitation, or occupational therapist (3)
- ▢ Other (please describe) (4) ____
- ▢ None (5)
- ▢ I don't know/don't remember (6)

Q78 **Have you had difficulty scheduling an appointment to follow-up about your injuries with… (select all that apply)**

- [ ] A trauma doctor, surgeon, or specialist (1)
- [ ] A family doctor (2)
- [ ] A physio, rehabilitation, or occupational therapist (3)
- [ ] Other (please describe) (4) ____
- [ ] None (5)
- [ ] I don't know/don't remember (6)

Display This Question:

If After being discharged from the hospital, have you attended an appointment to follow-up about you... = A trauma doctor, surgeon, or specialist

Or After being discharged from the hospital, have you attended an appointment to follow-up about you... = A family doctor

Or After being discharged from the hospital, have you attended an appointment to follow-up about you... = A physio, rehabilitation, or occupational therapist

Or After being discharged from the hospital, have you attended an appointment to follow-up about you... = Other (please describe)

Q17.4 **For the next 7 questions, “health care practitioners” means doctors, nurses, physiotherapists, occupational therapists, and other professionals helping in the care of your injuries.**

Display This Question:

If After being discharged from the hospital, have you attended an appointment to follow-up about you... = A trauma doctor, surgeon, or specialist

Or After being discharged from the hospital, have you attended an appointment to follow-up about you... = A family doctor

Or After being discharged from the hospital, have you attended an appointment to follow-up about you... = A physio, rehabilitation, or occupational therapist

Or After being discharged from the hospital, have you attended an appointment to follow-up about you... = Other (please describe)

Q17.5 **At your follow-up appointments, did your healthcare practitioners <u>explain the next steps in your recovery from your injury;</u> for example, activities you should or should not do, necessary medications, tests, treatments, or other follow-up appointments?**

○ Yes, and I got all the information I wanted (1)

○ Yes, but I wanted more information (2)

○ No (3)

○ I don't know/don't remember (4)

Display This Question:

If After being discharged from the hospital, have you attended an appointment to follow-up about you... = A trauma doctor, surgeon, or specialist

Or After being discharged from the hospital, have you attended an appointment to follow-up about you... = A family doctor

Or After being discharged from the hospital, have you attended an appointment to follow-up about you... = A physio, rehabilitation, or occupational therapist

Or After being discharged from the hospital, have you attended an appointment to follow-up about you... = Other (please describe)

Q79 **At your follow-up appointments, did your healthcare practitioners <u>explain approximately how long</u> it would take you to recover?**

- ◯ Yes, and I got all the information I wanted (1)
- ◯ Yes, but I wanted more information (2)
- ◯ No (3)
- ◯ I don't know/don't remember (4)

Display This Question:

If After being discharged from the hospital, have you attended an appointment to follow-up about you... = A trauma doctor, surgeon, or specialist

Or After being discharged from the hospital, have you attended an appointment to follow-up about you... = A family doctor

Or After being discharged from the hospital, have you attended an appointment to follow-up about you... = A physio, rehabilitation, or occupational therapist

Or After being discharged from the hospital, have you attended an appointment to follow-up about you... = Other (please describe)

Use Reusable Choices

Q17.6 **At your follow-up appointments, <u>how often</u> did your healthcare practitioners <u>explain things about your injuries</u> in a way that you could <u>understand?</u>**

- ◯ Never (1)
- ◯ Sometimes (2)
- ◯ Usually (3)
- ◯ Always (4)
- ◯ I don't remember/don't know (5)

Display This Question:

If After being discharged from the hospital, have you attended an appointment to follow-up about you... = A trauma doctor, surgeon, or specialist

Or After being discharged from the hospital, have you attended an appointment to follow-up about you... = A family doctor

Or After being discharged from the hospital, have you attended an appointment to follow-up about you... = A physio, rehabilitation, or occupational therapist

Or After being discharged from the hospital, have you attended an appointment to follow-up about you... = Other (please describe)

X→

Q17.7 **At your follow-up appointments, when you or your parents expressed <u>concerns</u> or <u>frustrations</u>, <u>how often</u> did your healthcare practitioners <u>take action to deal with them</u>?**

- Never (1)
- Sometimes (2)
- Usually (3)
- Always (4)
- Neither my parents nor I had concerns or frustrations at my follow-up appointments (5)
- I don't remember/don't know (6)

Display This Question:

If After being discharged from the hospital, have you attended an appointment to follow-up about you... = A trauma doctor, surgeon, or specialist

Or After being discharged from the hospital, have you attended an appointment to follow-up about you... = A family doctor

Or After being discharged from the hospital, have you attended an appointment to follow-up about you... = A physio, rehabilitation, or occupational therapist

Or After being discharged from the hospital, have you attended an appointment to follow-up about you... = Other (please describe)

And What grade in school were you when you were injured? != Was not enrolled in school at the time

Use Reusable Choices

X→ X→

Q17.8 **At your follow-up appointments, were school attendance and/or performance issues specifically addressed by any of the healthcare practitioners?**

- Never (1)
- Sometimes (2)
- Usually (3)
- Always (4)
- I don't remember/don't know (5)
- Schoolwork was not a concern (6)

Display This Question:

If After being discharged from the hospital, have you attended an appointment to follow-up about you... = A trauma doctor, surgeon, or specialist

Or After being discharged from the hospital, have you attended an appointment to follow-up about you... = A family doctor

Or After being discharged from the hospital, have you attended an appointment to follow-up about you... = A physio, rehabilitation, or occupational therapist

Or After being discharged from the hospital, have you attended an appointment to follow-up about you... = Other (please describe)

And What grade in school were you when you were injured? != Was not enrolled in school at the time

Use Reusable Choices

Q17.9 **At your follow-up appointments, how often did your healthcare practitioners ask if you required <u>extra services for schoolwork</u> (i.e. someone to write for you, extra time on tests), related to your injuries?**

- ○ Never (1)
- ○ Sometimes (2)
- ○ Usually (3)
- ○ Always (4)
- ○ I don't remember/don't know (5)
- ○ Schoolwork was not a concern (6)

End of Block: Follow-up block

Start of Block: Discharge cont'd

Q80 **Did your family physician, pediatrician, or general practitioner receive information from the hospital about your injures, your hospital stay, or the care you would need to continue your recovery?**

- ○ No (1)
- ○ Yes, but they wanted more information (2)
- ○ Yes, and they got all the information they wanted (3)
- ○ I haven't seen a family physician, pediatrician, or general practitioner since being discharged (4)
- ○ I don't remember/don't know (5)

End of Block: Discharge cont'd

Start of Block: Overall Care

Q19.1 **Overall, how well were you guided through your recovery process by your healthcare practitioners after being discharged from the hospital (on a scale of zero to ten, zero being poor guidance, ten being excellent guidance)?**

- ○ 0 Poor Guidance (1)
- ○ 1 (2)
- ○ 2 (3)
- ○ 3 (4)
- ○ 4 (5)
- ○ 5 (6)
- ○ 6 (7)
- ○ 7 (8)
- ○ 8 (9)
- ○ 9 (10)
- ○ 10 Excellent Guidance (11)

Q19.2 **On a scale of zero to ten, what is your overall rating of the follow-up care you received after being discharged from the hospital (zero being the worst injury care possible, ten being the best)?**

- ○ 0 (Worst Injury Care Possible) (1)
- ○ 1 (2)
- ○ 2 (3)
- ○ 3 (4)
- ○ 4 (5)
- ○ 5 (6)
- ○ 6 (7)
- ○ 7 (8)
- ○ 8 (9)
- ○ 9 (10)
- ○ 10 (Best Injury Care Possible) (11)

Q19.3 **What was the best aspect of the care you received for your injuries, either while in hospital or after discharge? You can list one or more things or say nothing**

____

Q19.4 **What was the worst aspect of the care you received for your injuries, either while in hospital or after discharge? You can list one or more things or say nothing**

____

End of Block: Overall Care

Start of Block: Reimbursement

Q20.1 What type of giftcard would you like?

- ○ Starbucks (1)
- ○ iTunes (2)
- ○ Best Buy (4)
- ○ Tim Hortons (5)
- ○ Chapters (6)
- ○ Cineplex (7)

Q20.2 Thank you for participating in our research! Your contribution will help improve the care of other individuals like yourself. Your giftcard should be emailed to the email address provided on the consent forms. ***Please click the arrow at the bottom right of your screen to submit your survey.***

End of Block: Reimbursement

Start of Block: Block 18

Q21.1 Thank you for your participation.

End of Block: Block 18
