# Supplementary material for: Development of the quality of teen trauma acute care patient and parent-reported experience measure
Source: BMC Res Notes. 2022 Sep 23;15:304. doi: 10.1186/s13104-022-06194-x (PMC9503226; doi:10.1186/s13104-022-06194-x)
Supplement: Supplementary file 2 — Additional file 2. QTTAC-PREOM Parent SF. [file 13104_2022_6194_MOESM2_ESM.pdf]

QTTAC-PREOM Parent SF

Start of Block: Survey Intro

Q1.1
We are doing a research study on teens who got injured and were admitted to the Foothills Medical Centre or the Alberta Children's Hospital in Calgary over the past year. Our hospital records show that your teen was among them. We are interested in finding out what you thought of the care they received in hospital and after they were discharged from hospital.

We understand that these injuries occurred some time ago, so we know that some answers may be hard to remember, and that is OK. We are interested in knowing what you think now, as you reflect back on the experience.

You don't have to agree to do this survey if you don't want to. We hope you do, however, as we want to use the results of this study to find ways to improve the care of other teens who get seriously injured and have to be hospitalized.

There are about 60 multiple-choice questions, and we have made them as simple as possible. It should take approximately 25 minutes or less. If there is a question you are unsure of the answer, there will be an option to reply "I don't know" or "not applicable." Though it is encouraged, you do not have to complete the entire survey at once. Survey completion is indicated at the end of the survey by text reading "thank you for your participation."

All of your answers will be kept strictly confidential and will only be used for the purpose of this research project. No one except you and the researchers will know you participated.

Q1.2
If you are still okay with going ahead, then please choose "Start survey." If you don't want to participate, then choose "I don't want to participate."

Click the arrow at the bottom right of your screen to proceed.

- ○ Start survey (1)
- ○ I don't want to participate (2)

End of Block: Survey Intro

Start of Block: Randomizer init

Q2.1
Thanks for agreeing to participate in this survey.

By agreeing to do so, this indicates to us that you understand the purpose of the survey and agree to let us use your answers to help find ways we can improve the care of teens who get major injuries and have to be hospitalized.

At the end of the survey, we will ask you for permission to send you an e-gift card as a token of thanks for helping us with this study.

Lets get started!

Click the arrow at the bottom right of your screen to proceed.

End of Block: Randomizer init

Start of Block: Demographics

Q3.1 We would like to know a little bit more about you. These questions are important to identify population-level trends. You may skip these questions if you wish not to answer.

Q3.2
What is <u>your teen's</u> ethnicity?

- ○ Caucasian (1)
- ○ Black or African Canadian (2)
- ○ Asian (3)
- ○ Aboriginal or First Nations (4)
- ○ East Indian (5)
- ○ Latin American (6)
- ○ Other (please indicate) (7) ____

Q3.3
What language do <u>you</u> mainly speak at home?

- ○ English (1)
- ○ French (2)
- ○ Vietnamese (3)
- ○ Spanish (4)
- ○ Chinese (Mandarin or Cantonese) (5)
- ○ Other (please indicate) (6) ____

Q3.4 What language does <u>your teen</u> mainly speak at home?

- ◯ English (1)
- ◯ French (2)
- ◯ Vietnamese (3)
- ◯ Spanish (4)
- ◯ Chinese (Mandarin or Cantonese) (5)
- ◯ Other (please indicate) (6) ____

Q3.5 Are you his/her

- ◯ Mother (1)
- ◯ Father (2)
- ◯ Other (please indicate) (3) ____

Q3.6 What is the highest level of education <u>you</u> have completed?

- ◯ 8th grade or less (1)
- ◯ Some high school, but did not graduate (2)
- ◯ High school graduate (3)
- ◯ Some college/university, did not graduate (4)
- ◯ College diploma/certificate (5)
- ◯ 4 year university degree (6)
- ◯ More than 4 year university degree (MSc, PhD, MD, etc.) (7)

Q3.7 Does your teen live with you...

- ◯ Almost never (1)
- ◯ Some of the time (2)
- ◯ Most of the time (3)
- ◯ All of the time (4)

End of Block: Demographics

Q6.1
You are now beginning the Quality of Teen Trauma Care Parent Reported Experience Measure (QTTAC-PREM).

The following questions are about your teen's experience while they were hospitalized, and your teen's experience after they left the hospital.

End of Block: QTAC PREOM Start

Start of Block: Caregiver Accommodation

Q8.1 **Which best describes the overnight accommodation you used most during your teen's hospital stay?**

- ◯ Your own home (1)
- ◯ Home of relatives or friends (2)
- ◯ Hotel or motel (3)
- ◯ Room provided by Ronald McDonald House (4)
- ◯ Room provided by other charitable organization (5)
- ◯ Separate room provided by hospital (6)
- ◯ Same room as my teen (7)

Q8.2 **Were accommodations available for you/the teen's caregiver to <u>stay with or near your teen</u> (e.g. down the hall, a bed in your teen's hospital room)?**

- ○ Yes (1)
- ○ No (2)
- ○ I don't know/don't remember (3)

Page Break

Use Reusable Choices

X→ X→

Q8.3 **During your teen's stay in hospital, did you or another caregiver <u>stay overnight</u> with or near your teen?**

- Never (1)
- Sometimes (2)
- Usually (3)
- Always (4)
- I don't remember/don't know (5)

End of Block: Caregiver Accommodation

Start of Block: Friends block

Use Reusable Choices

X→ X→

Q9.1 **How often did your teen's friends visit them while they were in hospital?**

- Never (1)
- Sometimes (2)
- Usually (3)
- Always (4)
- I don't remember/don't know (5)

Display This Question:

If If How often did your teen's friends visit them while they were in hospital? Never Is Selected

Q9.2 **Why do you think your teen's friends did not visit them in hospital (select all that apply)?**

- ▢ My teen did not stay in hospital very long (1)
- ▢ My teen's friends live far from the hospital (2)
- ▢ My teen told them not to visit (3)
- ▢ They felt uncomfortable visiting my teen (4)
- ▢ Other (please describe) (5) ____
- ▢ I don't remember/don't know (6)

Display This Question:

If If How often did your teen's friends visit them while they were in hospital? Never Is Selected

Or Or How often did your teen's friends visit them while they were in hospital? I don't remember/don't know Is Selected

Use Reusable Choices

Q9.3 **How often do you think <u>your teen's friends would have felt comfortable</u> visiting them while they were in hospital?**

- ○ Never (1)
- ○ Sometimes (2)
- ○ Usually (3)
- ○ Always (4)
- ○ I don't remember/don't know (5)

Display This Question:

If If How often did your teen's friends visit them while they were in hospital? Never Is Selected

Or Or How often did your teen's friends visit them while they were in hospital? I don't remember/don't know Is Selected

Use Reusable Choices

Q9.4 **How often would your teen have felt comfortable having their friends visit them while they were in the hospital?**

- Never (1)
- Sometimes (2)
- Usually (3)
- Always (4)
- I don't remember/don't know (5)

Display This Question:

If If How often did your teen's friends visit them while they were in hospital? Never Is Not Selected

And And How often did your teen's friends visit them while they were in hospital? I don't remember/don't know Is Not Selected

Use Reusable Choices

Q9.5 **How often do you think your teen's friends felt comfortable visiting them while they were in hospital?**

- Never (1)
- Sometimes (2)
- Usually (3)
- Always (4)
- I don't remember/don't know (5)

Display This Question:

If If How often did your teen's friends visit them while they were in hospital? Never Is Not Selected

And And How often did your teen's friends visit them while they were in hospital? I don't remember/don't know Is Not Selected

Use Reusable Choices

Q9.6 **How often do you think your teen felt comfortable having their friends visit them while they were in the hospital?**

- Never (1)
- Sometimes (2)
- Usually (3)
- Always (4)
- I don't remember/don't know (5)

Display This Question:

If If How often do you think your teen's friends felt comfortable visiting them while they were in hosp... Never Is Selected

Or Or How often do you think your teen's friends felt comfortable visiting them while they were in hosp... Sometimes Is Selected

Or Or How often do you think your teen's friends felt comfortable visiting them while they were in hosp... Usually Is Selected

Or Or How often do you think your teen's friends felt comfortable visiting them while they were in hosp... Always Is Selected

Q9.7 **Why do you feel your teen's friends were not comfortable visiting them while they were in hospital?**

____

Display This Question:

If If How often do you think your teen felt comfortable having their friends visit them while they were... Never Is Selected

Or Or How often do you think your teen felt comfortable having their friends visit them while they were... Sometimes Is Selected

Or Or How often do you think your teen felt comfortable having their friends visit them while they were... Usually Is Selected

Or Or How often do you think your teen felt comfortable having their friends visit them while they were... Always Is Selected

Q9.8 **Why do you feel <u>your teen</u> was <u>not comfortable</u> having their friends visit them while they were in hospital?**

____

End of Block: Friends block

Start of Block: Friends Block Cont'd

X→

Q10.1 **How did your teen <u>communicate with their friends</u> during their stay in hospital, <u>the majority of the time?</u>**

- ◯ In-person (1)
- ◯ Telephone conversations (2)
- ◯ Texting (3)
- ◯ Social media (Instagram, Snapchat, etc.) (4)
- ◯ They were unable to communicate with friends while in hospital (5)
- ◯ Other (please explain) (6) ____
- ◯ I don't remember/don't know (7)

End of Block: Friends Block Cont'd

Start of Block: School Assistance Block

Q12.1 **What grade in school was your teen in when they were injured?**

- 8th grade or less (1)
- Grade 9 (2)
- Grade 10 (3)
- Grade 11 (4)
- Grade 12 (5)
- 1st year University or above (6)
- Trade school (7)
- Was not enrolled in school at the time (8)
- I don't remember/don't know (9)

Q18.3 **During your** teen's **hospitalization, approximately how many days of school did your teen miss?**

- 0-5 (1)
- 6-10 (2)
- 11-15 (3)
- 16-35 (4)
- They are not back at school (5)
- They were not enrolled in school (6)
- I don't know/don't remember (7)

Display This Question:

If What grade in school was your teen in when they were injured? != Was not enrolled in school at the time

Use Reusable Choices

Q12.2 **How often did your** teen's **healthcare practitioners help them to keep up with schoolwork while in hospital?**

- Never (1)
- Sometimes (2)
- Usually (3)
- Always (4)
- I don't remember/don't know (5)
- Schoolwork was not a concern (6)

Display This Question:

If What grade in school was your teen in when they were injured? != Was not enrolled in school at the time

Use Reusable Choices

Q12.3 **How often did your teen's healthcare practitioners ask if they required <u>supplemental services for schoolwork</u> related to their injuries(i.e. someone to write for them, extra time on tests)?**

- ○ Never (1)
- ○ Sometimes (2)
- ○ Usually (3)
- ○ Always (4)
- ○ I don't remember/don't know (5)
- ○ Schoolwork was not a concern (6)

End of Block: School Assistance Block

Start of Block: Default Question Block

Q7.1 **When <u>meeting new healthcare practitioners</u> for the first time, how often did they <u>introduce themselves</u> and clearly e<u>xplain their role</u> in your teen's care?**

- ○ Never (1)
- ○ Sometimes (2)
- ○ Usually (3)
- ○ Always (4)
- ○ I don't remember/don't know (5)

Q7.2 **How often did your teen's healthcare practitioners clearly explain all their injuries to you in a way you could understand?**

- Never (1)
- Sometimes (2)
- Usually (3)
- Always (4)
- I don't remember/don't know (5)

Q7.3 **How often did the healthcare practitioners (e.g. doctors, nurses, therapists, etc.) <u>explain your teen's treatment</u> in a way you could <u>understand</u>?**

- Never (1)
- Sometimes (2)
- Usually (3)
- Always (4)
- I don't know/don't remember (5)

Q14.2 **Did the healthcare practitioners give <u>instructions</u> on how you should <u>care for your teen's injuries</u>?**

- Yes, we received all the instruction we needed (1)
- Yes, but we only received some of the instruction we needed (2)
- No (3)
- I don't remember/don't know (4)

Q7.4 **Did the healthcare practitioners discuss <u>how long</u> it might take your teen <u>to recover</u> from their injuries?**

- ◯ Yes (1)
- ◯ No (2)
- ◯ I don't know/don't remember (3)

Q7.5 **Did the healthcare practitioners discuss <u>the long-term consequences of your</u>** <u>teen's</u> **<u>injuries</u> (on sports, music, extracurriculars, etc.) after they leave the hospital?**

- ◯ Yes (1)
- ◯ No (2)
- ◯ I don't know/don't remember (3)

Use Reusable Choices

X→ X→

Q7.6 **How often was the information that was given by your teen's various healthcare practitioners <u>consistent</u>?**

- ◯ Never (1)
- ◯ Sometimes (2)
- ◯ Usually (3)
- ◯ Always (4)
- ◯ I don't remember/don't know (5)

End of Block: Default Question Block

Start of Block: Block 4

Q93 **When the healthcare practitioners helped your teen to <u>move around</u> (i.e., change position in bed, walking etc.) how often did they do it carefully?**

- ○ Never (1)
- ○ Sometimes (2)
- ○ Usually (3)
- ○ Always (4)
- ○ My teen did not need help moving (5)
- ○ I don't remember/don't know (6)

Q11.4 **How often was your teen's <u>pain from their injuries well controlled</u>?**

- ○ Never (1)
- ○ Sometimes (2)
- ○ Usually (3)
- ○ Always (4)
- ○ My teen had no pain (5)
- ○ I don't remember/don't know (6)

Q11.5 **How often did the healthcare practitioners do everything they could to help your teen with their discomfort, agitation or irritability?**

- ○ Never (1)
- ○ Sometimes (2)
- ○ Usually (3)
- ○ Always (4)
- ○ My teen had no feelings of agitation or irritability (5)
- ○ I don't remember/don't know (6)

Q11.6 **When you or your teen had questions, concerns or frustrations about your teen's care, how often did their healthcare practitioners take action?**

- ○ Never (1)
- ○ Sometimes (2)
- ○ Usually (3)
- ○ Always (4)
- ○ Neither my teen or I had any questions, concerns or frustrations about his/her care (5)
- ○ I don't remember/don't know (6)

Q11.7 **Did a healthcare practitioner (e.g. psychologist, social worker, nurse) <u>offer</u> to <u>speak</u> to you or your teen about their <u>mental or emotional health</u>?**

- ◯ Yes, and they got all the support they needed (1)
- ◯ Yes, but they needed more support (2)
- ◯ Yes, but they did not need support (3)
- ◯ No, and they felt they needed support (4)
- ◯ No, but they did not need support (5)
- ◯ I don't remember/don't know (6)

Use Reusable Choices

Q11.8 **How often did the hospital staff <u>offer to help your teen</u> maintain their <u>personal hygiene (brushing teeth, bathing, etc)</u>?**

- ◯ Never (1)
- ◯ Sometimes (2)
- ◯ Usually (3)
- ◯ Always (4)
- ◯ I don't remember/don't know (5)

Use Reusable Choices

Q11.9 **Did you have privacy in your teen's hospital room?**

- Never (1)
- Sometimes (2)
- Usually (3)
- Always (4)
- I don't remember/don't know (5)

Use Reusable Choices

Q11.10 **How often did your teen experience care that you thought was unsafe?**

- Never (1)
- Sometimes (2)
- Usually (3)
- Always (4)
- I don't remember/don't know (5)

Use Reusable Choices

Q11.11 **How often was your teen treated <u>unfairly</u> because of their <u>age, ethnicity, gender, or personal characteristics</u>?**

- ◯ Never (1)
- ◯ Sometimes (2)
- ◯ Usually (3)
- ◯ Always (4)
- ◯ I don't remember/don't know (5)

Use Reusable Choices

Q11.12 **How often did you feel your teen was treated in a way that was <u>not appropriate for their age</u>?**

- ◯ Never (1)
- ◯ Sometimes (2)
- ◯ Usually (3)
- ◯ Always (4)
- ◯ I don't remember/don't know (5)

End of Block: Block 4

Start of Block: Overall care

Q13.1 **Please provide an overall rating, between 0 and 10, of the hospital care they received for their injury (with 0 being the worst care possible and 10 being the best)?**

- ◯ 0 Worst Injury Care Possible (1)
- ◯ 1 (2)
- ◯ 2 (3)
- ◯ 3 (4)
- ◯ 4 (5)
- ◯ 5 (6)
- ◯ 6 (7)
- ◯ 7 (8)
- ◯ 8 (9)
- ◯ 9 (10)
- ◯ 10 Best injury Care Possible (11)

End of Block: Overall care

Start of Block: Pre-Discharge Care

Q14.1 **Where did your teen go after being discharged from the Alberta Children's Hospital/Foothills Medical Centre?**

- ◯ Home (either yours or someone else's) (1)
- ◯ Another hospital (2)
- ◯ An in-patient rehabilitation facility (3)
- ◯ A long-term care facility (4)

Display This Question:

If Where did your teen go after being discharged from the Alberta Children's Hospital/Foothills Medi... = Home (either yours or someone else's)

Q14.3 **Before leaving the hospital, did your teen's *doctors or nurses* give you or another caregiver <u>written instructions</u> on <u>how to care for their injuries</u> after being discharged?**

- ◯ Yes (1)
- ◯ No (2)
- ◯ I don't remember/don't know (3)

Display This Question:

If Before leaving the hospital, did your teen's doctors or nurses give you or another caregiver wri... = Yes

Q14.4 **Did the written instructions provided give him/her <u>enough information</u> to <u>help</u> him/her to care for his/her injuries after being discharged?**

- ◯ Yes (1)
- ◯ No (2)
- ◯ I don't remember/don't know (3)

End of Block: Pre-Discharge Care

Start of Block: Post-discharge block

Q15.1 **After your teen's stay in hospital…**

End of Block: Post-discharge block

Start of Block: Medications block

Q16.1 **After being discharged from the hospital, did *your teen* have <u>enough pain medication</u> to <u>control his/her pain well</u>?**

- ◯ Yes (1)
- ◯ No (2)
- ◯ My teen didn't need any pain medication (3)
- ◯ I don't remember/don't know (4)

Display This Question:

If After being discharged from the hospital, did your teen have enough pain medication to control h... = Yes

Or After being discharged from the hospital, did your teen have enough pain medication to control h... = No

Or After being discharged from the hospital, did your teen have enough pain medication to control h... = I don't remember/don't know

Q16.2 **After being discharged from the hospital, did *your teen* <u>receive a prescription for opioids to control his/her pain?</u> (Ex. Tramadol, Dilaudid, Ultram, etc.)**

- ◯ Yes (1)
- ◯ No (2)
- ◯ I don't remember/don't know (3)

Display This Question:

If After being discharged from the hospital, did your teen receive a prescription for opioids to c... = Yes

Q16.3 **After being discharged from the hospital, did *your teen* <u>fill out your prescription for opioids to control his/her pain?</u> (Ex. Tramadol, Dilaudid, Ultram, etc.)**

- ◯ Yes (1)
- ◯ No (2)
- ◯ I don't remember/don't know (3)

End of Block: Medications block

Start of Block: Post-discharge support services

Q17.1 **After being discharged from the hospital, did *your teen* get <u>all of the support services that they wanted or felt they needed</u>? (for example, home care, social work, or counselling)**

- Yes (1)
- No (2)
- I don't know/don't remember (3)

Q17.2 **After being discharged, to what extent was your teen able to maintain his/her friends/social network?**

- Never (1)
- Sometimes (2)
- Usually (3)
- Always (4)
- I don't know/don't remember (5)

End of Block: Post-discharge support services

Start of Block: Follow-up block

Q18.1 **After being discharged from the hospital, did *your teen* <u>attend</u> any appointments to follow-up about their injuries with… (select all that apply)**

- ☐ A trauma doctor, surgeon, or specialist (1)
- ☐ A family doctor (2)
- ☐ A physio, rehabilitation, or occupational therapist (3)
- ☐ Other (please describe) (4) ____
- ☐ None (5)
- ☐ I don't know/don't remember (6)

Q87 **Have you or your teen scheduled or are planning to schedule an appointment to follow-up about their injuries with… (select all that apply)**

- ☐ A trauma doctor, surgeon, or specialist (1)
- ☐ A family doctor (2)
- ☐ A physio, rehabilitation, or occupational therapist (3)
- ☐ Other (please describe) (4) ____
- ☐ None (5)
- ☐ I don't know/don't remember (6)

Q18.2 **Did *you* have any <u>difficulty scheduling follow-up appointments for your teen</u> when either of you wanted them with... (select all that apply)**

- ☐ A trauma doctor, surgeon, or specialist (1)
- ☐ A family doctor (2)
- ☐ A physio, rehabilitation, or occupational therapist (3)
- ☐ Other (please describe) (4) ____
- ☐ None (5)
- ☐ I don't know/don't remember (6)

Display This Question:

If After being discharged from the hospital, did your teen attend any appointments to follow-up abou... = A trauma doctor, surgeon, or specialist

Or After being discharged from the hospital, did your teen attend any appointments to follow-up abou... = A family doctor

Or After being discharged from the hospital, did your teen attend any appointments to follow-up abou... = A physio, rehabilitation, or occupational therapist

Or After being discharged from the hospital, did your teen attend any appointments to follow-up abou... = Other (please describe)

Q18.4 **For the next 7 questions, “healthcare practitioner” means doctors, nurses, physiotherapists, occupational therapists, and other professionals helping in the care of your** teen's **injuries.**

Display This Question:

If After being discharged from the hospital, did your teen attend any appointments to follow-up abou... = A trauma doctor, surgeon, or specialist

Or After being discharged from the hospital, did your teen attend any appointments to follow-up abou... = A family doctor

Or After being discharged from the hospital, did your teen attend any appointments to follow-up abou... = A physio, rehabilitation, or occupational therapist

Or After being discharged from the hospital, did your teen attend any appointments to follow-up abou... = Other (please describe)

Q18.5 **At your teen's follow-up appointments, did your healthcare practitioners <u>explain the next steps in your teen's recovery from his/her injury;</u> for example, activities they should or should not do, necessary medications, tests, treatments, or other follow-up appointments?**

- ○ Yes, and I got all the information I wanted (1)
- ○ Yes, but I wanted more information (2)
- ○ No (3)
- ○ I don't know/don't remember (4)

Display This Question:

If After being discharged from the hospital, did your teen attend any appointments to follow-up abou... = A trauma doctor, surgeon, or specialist

Or After being discharged from the hospital, did your teen attend any appointments to follow-up abou... = A family doctor

Or After being discharged from the hospital, did your teen attend any appointments to follow-up abou... = A physio, rehabilitation, or occupational therapist

Or After being discharged from the hospital, did your teen attend any appointments to follow-up abou... = Other (please describe)

Q88 **At your teen's follow-up appointments, did their healthcare practitioners explain approximately how long it would take them to recover?**

- ◯ Yes, and I got all the information I wanted (1)
- ◯ Yes, but I wanted more information (2)
- ◯ No (3)
- ◯ I don't know/don't remember (4)

Display This Question:

If After being discharged from the hospital, did your teen attend any appointments to follow-up abou... = A trauma doctor, surgeon, or specialist

Or After being discharged from the hospital, did your teen attend any appointments to follow-up abou... = A family doctor

Or After being discharged from the hospital, did your teen attend any appointments to follow-up abou... = A physio, rehabilitation, or occupational therapist

Or After being discharged from the hospital, did your teen attend any appointments to follow-up abou... = Other (please describe)

Use Reusable Choices

Q18.6 **At your teen's follow-up appointments, how often did your healthcare practitioners explain things about his/her injuries in a way that you and your teen could understand?**

- ◯ Never (1)
- ◯ Sometimes (2)
- ◯ Usually (3)
- ◯ Always (4)
- ◯ I don't remember/don't know (5)

Display This Question:

If After being discharged from the hospital, did your teen attend any appointments to follow-up abou... = A trauma doctor, surgeon, or specialist

Or After being discharged from the hospital, did your teen attend any appointments to follow-up abou... = A family doctor

Or After being discharged from the hospital, did your teen attend any appointments to follow-up abou... = A physio, rehabilitation, or occupational therapist

Or After being discharged from the hospital, did your teen attend any appointments to follow-up abou... = Other (please describe)

X→

Q18.7 **At your teen's follow-up appointments, when you or your teen expressed concerns or frustrations, how often did his/her healthcare practitioners take action to deal with them?**

- Never (1)
- Sometimes (2)
- Usually (3)
- Always (4)
- Neither my teen nor I expressed concerns or frustrations at my follow-up appointments (5)
- I don't remember/don't know (6)

Display This Question:

If After being discharged from the hospital, did your teen attend any appointments to follow-up abou... = A trauma doctor, surgeon, or specialist

Or After being discharged from the hospital, did your teen attend any appointments to follow-up abou... = A family doctor

Or After being discharged from the hospital, did your teen attend any appointments to follow-up abou... = A physio, rehabilitation, or occupational therapist

Or After being discharged from the hospital, did your teen attend any appointments to follow-up abou... = Other (please describe)

And What grade in school was your teen in when they were injured? != Was not enrolled in school at the time

Use Reusable Choices

X→ X→

Q18.8 **At your teen's follow-up appointments, were school attendance and/or performance issues specifically addressed by any of the healthcare practitioners?**

- ◯ Never (1)
- ◯ Sometimes (2)
- ◯ Usually (3)
- ◯ Always (4)
- ◯ I don't remember/don't know (5)
- ◯ Schoolwork was not a concern (6)

Display This Question:

If After being discharged from the hospital, did your teen attend any appointments to follow-up abou... = A trauma doctor, surgeon, or specialist

Or After being discharged from the hospital, did your teen attend any appointments to follow-up abou... = A family doctor

Or After being discharged from the hospital, did your teen attend any appointments to follow-up abou... = A physio, rehabilitation, or occupational therapist

Or After being discharged from the hospital, did your teen attend any appointments to follow-up abou... = Other (please describe)

And What grade in school was your teen in when they were injured? != Was not enrolled in school at the time

Use Reusable Choices

Q18.9 **At your teen's follow-up appointments, how often did your teen's healthcare practitioners ask if they required <u>extra services for schoolwork</u> (i.e. someone to write for them, extra time on tests), related to their injuries?**

- ○ Never (1)
- ○ Sometimes (2)
- ○ Usually (3)
- ○ Always (4)
- ○ I don't remember/don't know (5)
- ○ Schoolwork was not a concern (6)

End of Block: Follow-up block

Start of Block: Discharge cont'd

Q89 **Did your teen's family physician, pediatrician, or general practitioner receive information from the hospital about your injures, your hospital stay, or the care you would need to continue your recovery?**

- ○ No (1)
- ○ Yes, but they wanted more information (2)
- ○ Yes, and they got all the information they wanted (3)
- ○ My teen hasn't seen a family physician, pediatrician, or general practitioner since being discharged (4)
- ○ I don't remember/don't know (5)

End of Block: Discharge cont'd

Start of Block: Overall Care

Q20.1 **Overall, how well were you and *your teen* <u>guided through the recovery process</u> by his/her healthcare practitioners <u>after being discharged from the hospital</u> on a scale of zero to ten (zero being poor guidance, ten being excellent guidance)?**

- ○ 0 Poor Guidance (1)
- ○ 1 (2)
- ○ 2 (3)
- ○ 3 (4)
- ○ 4 (5)
- ○ 5 (6)
- ○ 6 (7)
- ○ 7 (8)
- ○ 8 (9)
- ○ 9 (10)
- ○ 10 Excellent Guidance (11)

Q20.2 **On a scale of zero to ten, please provide an overall rating of the follow-up care *your teen* received after being discharged from the hospital (zero being the worst injury care possible, ten being the best)?**

- 0 (Worst Injury Care Possible) (1)
- 1 (2)
- 2 (3)
- 3 (4)
- 4 (5)
- 5 (6)
- 6 (7)
- 7 (8)
- 8 (9)
- 9 (10)
- 10 (Best Injury Care Possible) (11)

Q20.3 **What was the best aspect of the care your teen received for their injuries, either while in hospital or after discharge? You can list one or more things, or say nothing**

____

Q20.4 **What was the worst aspect of the care you received for their injuries, either while in hospital or after discharge? You can list one or more things, or say nothing**

____

End of Block: Overall Care

Start of Block: Reimbursement

Q21.1 What type of giftcard would you like?

- ○ Starbucks (1)
- ○ iTunes (2)
- ○ Best Buy (4)
- ○ Tim Hortons (5)
- ○ Cineplex (6)
- ○ Chapters (7)

Q21.2
Thank you for participating in our research! Your contribution will help improve the care of other individuals like yourself. Your giftcard should be emailed to the email address provided on the consent forms. ***Please click the arrow at the bottom right of your screen to submit your survey.***

End of Block: Reimbursement

Start of Block: Block 18

Q22.1 Thank you for your participation.

End of Block: Block 18
